# Liver Disease in Sri Lanka

**DOI:** 10.5005/jp-journals-10018-1217

**Published:** 2017-05-05

**Authors:** Hasitha S Wijewantha

**Affiliations:** Department of Gastroenterology and Hepatology, Provincial General Hospital, Badulla, Sri Lanka

**Keywords:** Causes of cirrhosis, Liver disease, Nonalcoholic fatty liver disease, Viral hepatitis.

## Abstract

Liver disease in Sri Lanka is mainly due to alcoholic liver disease and nonalcoholic fatty liver disease. In contrast to other South Asian countries, the prevalence of hepatitis B and C is low in Sri Lanka and prevalence of hepatitis A is intermediate. The few reported cases of hepatitis E in Sri Lanka are mainly in people who have traveled to neighboring South Asian countries. Wilson’s disease, autoimmune hepatitis, hemochromatosis, drug-induced liver disease, and primary biliary cirrhosis are recognized causes of liver disease in Sri Lanka. Pyogenic and amebic liver abscesses and dengue infection are the other causes of liver disease. Some of the commonly used plants as traditional herbal medicine in Sri Lanka have been shown to have deleterious effects on the liver in animal studies. Considering the high popularity of traditional herbal medicine in the country, it is likely that herbal medicine is an etiological factor for liver disease in Sri Lanka, but no published data are available.

Address reprint requests to: Wijewantha HS. Liver Disease in Sri Lanka. Euroasian J Hepato-Gastroenterol 2017;7(1):78-81.

## INTRODUCTION

Sri Lanka is an island in the Indian Ocean situated close to the southern tip of India. It has a population of just over 20 million of which the majority are Sinhalese (74.9%). Sri Lankan Tamil (11.2%), Sri Lankan Moor (9.3%), and Indian Tamil (4.1%)^1^ are the other minor ethnic groups in the Island, which spans for 65600 km^2^. Education and health are fully funded by the state and thus are free for all citizens in Sri Lanka. This has led to remarkably high literacy rate (92.3%) and life expectancy at birth (72 years for male and 78 years for females)^2^ when compared with other countries in the region and rest of the third world. Sri Lanka has an excellent network of primary, secondary, and tertiary care hospitals run by the government, which are distributed throughout the country to provide health care to almost all its citizens.

Liver diseases are generally given a lesser priority to many other communicable and noncommunicable diseases in Sri Lanka. However, from the practicing clinicians’ point of view, liver disease poses a significant health problem and is a major cause for morbidity and mortality. Two most common etiologies for liver disease in Sri Lanka are nonalcoholic fatty liver disease (NAFLD) and alcoholic liver disease. However, published data on the burden of liver disease in Sri Lanka are extremely sparse.

## NONALCOHOLIC FATTY LIVER DISEASE

Due to rising incidence of diabetes and metabolic syndrome, NAFLD has become the major cause of liver disease in Sri Lanka. A nationwide cross-sectional study conducted in 2005 to 2006 found that 21.8% of total population had some form of dysglycemia.^3^ Another study conducted in four provinces of Sri Lanka among adults between the ages of 30 and 65 years found 20.4% of males and 36.5% of females were obese (body mass index > 25 kg/m^2^).^4^ Study conducted in an urban area showed prevalence of NAFLD to be 32.6%, with 62.1% of affected being females.^5^ Unfortunately, no published data are available on national prevalence of NAFLD in Sri Lanka. However, it is evident that NAFLD is the commonest cause for consultations for deranged liver function tests and has overtaken alcoholic liver disease as a cause for chronic liver disease in Sri Lanka.

## ALCOHOLIC LIVER DISEASE

Unsafe alcohol use is a common cause for liver disease among males in Sri Lanka. Alcohol use is uncommon among females in Sri Lanka. A community-based study in Colombo district showed that only 3.7% of females had used alcohol 12 or more times in their lifetime. The same study found that 6.2% of men had a history of alcohol abuse and 4% of men had alcohol dependence.^6^ A retrospective study conducted in a hepatology clinic at Colombo North Teaching Hospital showed that 54.7% of patients with cirrhosis attending the clinic had an alcoholic etiology. Out of the patients with alcoholic cirrhosis, only 2.3% were females.^7^

## HEPATITIS A

Sri Lanka is a country with an intermediate prevalence of hepatitis A. The number of nonimmune adults susceptible for hepatitis A infection has increased over the past few decades. As a result, Sri Lanka experiences outbreaks of hepatitis A, which has become a significant health problem.^8^ Random blood sampling in healthy individuals living in Colombo in 1975 showed prevalence of hepatitis A immunoglobulin G to be 76.9%; age-specific analysis indicated that infections were acquired during child-hood.^9^ In 2001, a study conducted in Lady Ridgeway Children’s Hospital in Colombo among 288 children showed only 11.6% seroprevalence of hepatitis A antibody among children aged less than 10 years, and 78% of them were from families earning less than 10,000 rupees a month.^10^

## HEPATITIS B

Sri Lanka introduced hepatitis B vaccination into the extended program of immunization in 2003. It is given free of charge to all newborns through government health care institutions.^11^ There are no published data on island-wide prevalence of hepatitis B infection in Sri Lanka. Studies conducted on different population groups, including blood donors, pregnant mothers, and prison inmates, have shown prevalence of hepatitis B is less than 2%.^12^ Study conducted in a nonrepresentative sample of 25 patients with chronic hepatitis B revealed genotypes B, C, and D were the commonest genotypes.^13^ Despite its location in a region where prevalence of hepatitis B is high, hepatitis B infection is not common in Sri Lanka and is not a major health problem. A single-center retrospective study involving 696 patients with cirrhosis found only 13 (1.87%) patients had chronic hepatitis B infection.^7^ In another study done on 81 patients with cirrhosis who were referred for liver transplantation, none had hepatitis B or C.^14^

## HEPATITIS C

Even though nationwide data on prevalence of hepatitis C virus (HCV) in Sri Lanka are not available, studies conducted in various specific groups of population have shown a seroprevalence of <1%. Study involving 396 prison inmates in two major prisons in Sri Lanka showed HCV antibody positivity in 27 (6.9%) participants. Out of them, only 2 (0.5%) were positive for hepatitis C ribonucleic acid (RNA). This cohort consisted of 17 (4.3%) intravenous drug users and none of them were positive for hepatitis C.^12^ Another study, which analyzed 4,980 blood samples from healthy blood donors representing all districts of Sri Lanka, found that 53 (1.1%) samples were positive for HCV antibodies. Out of these, only 8 (0.2%) samples were positive for HCV RNA. On genotype analysis, seven samples were genotype 3a and one was genotype 3b.^15^ In another study involving 696 patients with cirrhosis, only 3 patients (0.4%) had HCV-related cirrhosis.^7^

## HEPATITIS E

Hepatitis E virus infection is rare in Sri Lanka and no data available on prevalence. The few reported cases of hepatitis E infection have been in patients who have visited other South Asian countries.

## HERBAL MEDICINE AND TOXINS

Use of traditional herbal medicine (Ayurveda) for ailments is a common practice in Sri Lanka. Alcohol is used as an ingredient in preparation of some of the traditional medications. However, its contribution to the burden of liver disease in the country is not known.

Feeding rats with material obtained from three commonly used medicinal plants in Sri Lanka, such as *Crotalaria verrucosa, Holarrhena antidysenterica,* and *Cassia auriculata,* resulted in disruption of the centrilobular veins, congestion or hemorrhage in the centrilobular sinusoids, and centrilobular or focal hepatocellular necro-sis.^16^ However, there are no studies on herbal medicine toxicity on humans.

There are no reported cases of aflatoxin-related liver disease in Sri Lanka. A study conducted in main rice-producing areas in Sri Lanka revealed parboiled and milled rice contained significantly higher content of anatoxin B1 (highest content 185 ug/kg) and anatoxin G1 (highest content 963 ug/kg) compared with raw milled rice.^17^ However, consumption of parboiled rice is much lesser than that of raw milled rice in Sri Lanka.

## PYOGENIC AND AMEBIC LIVER ABSCESSES

Liver abscesses are a relatively common problem in Sri Lanka; however, the exact prevalence is not known. There is a case report on a 14-year-old child presenting with a large abscess in the right lobe of the liver which was due to *Klebsiella* species.^18^ There is another case report of a patient presenting with multiple liver lesions after visiting Sri Lanka, which had been ultimately proven to be due to multiple amebic liver abscesses.^19^ Liver abscesses are a common problem, especially in rural areas of Sri Lanka.

**Table 1:** **Table 1:** Etioloav of Cirrhosis (adapted from Senanavke SM et al)^7^

*Etiology*		*Number*		*Percentage*	
Alcohol		381		54.74	
Cryptogenic		270		38.79	
Hepatitis B		13		1.87	
Wilson’s disease		13		1.87	
Autoimmune hepatitis		7		1.01	
Drug induced		4		0.57	
Hepatitis C		3		0.43	
Hemochromatosis		3		0.43	
Primary biliary cirrhosis		1		0.14	
Traditional and herbal medicine		1		0.14	
Total		696		100.00	

### LIVER DISEASE DUE TO DENGUE INFECTION

Dengue is one of the commonest infectious diseases in Sri Lanka, especially in urban areas. Over 45,000 cases of dengue have been reported in first 11 months of 2016.^20^ Liver involvement is common in severe dengue infection. Aspartate transaminase generally tends to be higher than alanine transaminase and sometimes transaminase levels can be >1000 IU. Cholestasis and biliary stasis are uncommon in dengue infection, and alkaline phosphatase and bilirubin levels are only minimally elevated. Dengue-associated liver failure is a condition with high mortality and is known to occur with or without evidence of shock.^21^

### OTHER CAUSES OF LIVER DISEASE IN SRI LANKA

Etiology of cirrhosis in 696 patients who attended a tertiary care hepatology clinic at Colombo North Teaching hospital from 1995 to 2010 is shown in Table 1.^7^

Due to lack of data, the exact population prevalence of autoimmune hepatitis, Wilson’s disease, primary biliary cirrhosis, and hemochromatosis in Sri Lanka is not known.

## References

[1] Department of Census and Statistics Sri Lanka. Census of population and housing 2012 - final report [Internet]. Colombo, Sri Lanka: Department of Census and Statistics of Sri Lanka; [published 2015 July 30; cited 2016 Nov 29]. Available from:. http://www.statistics.gov.lk/pophousat/cph2011/index.php?fileName=Activities/TentativelistofPublications..

[2] Medical statistics unit ministry of health nutrition and indigenous medicine. Annual health bulletin 2014 [Inter-net]. Colombo: Ministry of health nutrition and indigenous medicine of Sri Lanka; 2016 [cited 2016 Nov 29].221p.Available from:. http://www.health.gov.lk/moh_final/english/public/elfinder/files/publications/AHB/AHB2014pdf..

[3] Katulanda P, Constantine GR, Mahesh JG, Sheriff R, Seneviratne RD, Wijeratne S, Wijesuriya M, McCarthy MI, Adler AI, Matthews DR (2008). Prevalence and projections of diabetes and pre-diabetes in adults in Sri Lanka - Sri Lanka Diabetes, on Cardiovascular study (SLDCS). Diabet Med.

[4] Wijewardene K, Mohideen MR, Mendis S, Fernando DS, Kulathilaka T, Weerasekara D, Uluwitta P (2005). Prevalence of hypertension, diabetes and obesity: baseline findings of a population based survey in four provinces in Sri Lanka. Ceylon Med J.

[5] Dassanayake AS, Kasturiratne A, Rajindrajith S, Kalubowila U, Chakrawarthi S, De Silva AP, Makaya M, Mizoue T, Kato N, Wickremasinghe AR (2009). Prevalence and risk factors for non-alcoholic fatty liver disease among adults in an urban Sri Lankan population. J Gastroenterol Hepatol.

[6] Zavos HM, Siribaddana S, Ball HA, Lynskey MT, Sumathipala A, Rijsdijk FV, Hotopf M (2015). The prevalence and correlates of alcohol use and alcohol abuse disorders: a population based study in Colombo, Sri Lanka. BMC Psychiatry.

[7] Senanayake SM, Niriella MA, Weerasinghe SK, Kasturiratne A, de Alwis JP, de Silva AP, Dassanayake AS, de Silva HJ (2012). Survival of patients with alcoholic and cryptogenic cirrhosis without liver transplantation: a single center retrospective study. BMC Res Notes.

[8] Dahanayaka NJ, Kiyohara T, Agampodi SB, Samaraweera PK, Kulasooriya GK, Ranasinghe JC, Semage SN, Yoshizaki S, Wakita T, Ishii K (2016). Clinical features and transmission pattern of hepatitis A: an experience from a hepatitis A outbreak caused by two cocirculating genotypes in Sri Lanka. Am J Trop Med Hyg.

[9] Moritsugu Y, Miyamura K, Utagawa E, Kono R, de la Motte PU, Forbes CA (1979). Prevalence of antibody to hepatitis A virus in Sri Lanka. Asian J Infect Dis.

[10] de Silva KS, Weerasuriya DC, Peelawattage M, Fernando S (2005). Seroprevalence of hepatitis A antibodies in relation to social factors - a preliminary study. Ceylon Med J.

[11] Pieris, TSR. National Immunisation Program of Sri Lanka & Key Principals in Immunisation. Colombo: Epidemiology Unit Ministry of Health; 2011 [updated 2011 May 16; cited 2016 Nov 29]. Available from:. http://www.epid.gov.lk/web/index.php?option=com_content&view=article&id=137&Itemid=426&lang=en..

[12] Niriella MA, Hapangama A, Luke HP, Pathmeswaran A, Kuruppuarachchi KA, de Silva HJ (2015). Prevalence of hepatitis B and hepatitis C infections and their relationship to injectable drug use in a cohort of Sri Lankan prison inmates. Ceylon Med J.

[13] Manamperi A, Gunawardene NS, Wellawatta C, Abeyewick-reme W, de Silva HJ (2011). Hepatitis B virus (HBV) genotypes in a group of Sri Lankan patients with chronic infection. Trop Biomed.

[14] Siriwardana RC, Niriella MA, Liyanage CA, Wijesuriya SR, Gunathilaka B, Dassanayake AS, De Silva HJ (2013). Cryptogenic cirrhosis is the leading cause for listing for liver transplantation in Sri Lanka. Indian J Gastroenterol.

[15] Senevirathna D, Amuduwage S, Weerasingam S, Jayasinghe S, Fernandopulle N (2011). Hepatitis C virus in healthy blood donors in Sri Lanka. Asian J Transfus Sci.

[16] Arseculeratne SN, Gunatilaka AA, Panabokke RG (1981). Studies on medicinal plants of Sri Lanka: occurrence of pyrrolizidine alkaloids and hepatotoxic properties in some traditional medicinal herbs. J Ethnopharmacol.

[17] Bandara JM, Vithanege AK, Bean GA (1991). Occurrence of afla-toxins in parboiled rice in Sri Lanka. Mycopathologia.

[18] De Silva GNN, Pathirana K (2001). Pyogenic liver abscess. Sri Lanka J Child Health.

[19] Cavailloles FA, Mure A, Nasser H, Lecapitaine AL, Granier F (2014). Multiple liver amoebic abscesses detected on FDG PET/CT. Clin Nucl Med.

[20] Epidemiology Unit Ministry of Health. Dengue update [Internet]. Colombo, Sri Lanka: Epidemiology Unit, Ministry of Health; 2016 [updated 2016 Nov 29; cited 2016 Nov 30]. Available from:. http://www.epid.gov.lk/web/index.php?option=com_content&view=article&id=171%o3Adengue-update..

[21] Fernando S, Wijewickrama A, Gomes L, Punchihewa CT, Madusanka SD, Dissanayake H, Jeewandara C, Peiris H, Ogg GS, Malavige GN (2016). Patterns and causes of liver involvement in acute dengue infection. BMC Infect Dis.

